# Reporting of major complications in randomized clinical trials in pancreatic surgery according to Clavien–Dindo classification

**DOI:** 10.1093/bjsopen/zraf103

**Published:** 2025-09-09

**Authors:** Amila Cizmic, Laetitia Hampe, Philipp A Wise, Pascal Probst, Markus K Muller, Christoph Kuemmerli, Philip C Müller, Jan Bardenhagen, Anna Nießen, Faik G Uzunoglu, Jakob Izbicki, Thilo Hackert, Felix Nickel

**Affiliations:** Department of General, Visceral and Thoracic Surgery, University Medical Center Hamburg-Eppendorf, Hamburg, Germany; Department of General, Visceral and Thoracic Surgery, University Medical Center Hamburg-Eppendorf, Hamburg, Germany; Department of Neuroradiology at the Neurology Center, Heidelberg University Hospital, Heidelberg, Germany; Department of Surgery, Cantonal Hospital Thurgau, Frauenfeld, Switzerland; Department of Surgery, Cantonal Hospital Thurgau, Frauenfeld, Switzerland; Department of Surgery, Clarunis University Digestive Health Care Center, Basel, Switzerland; Department of Surgery, Clarunis University Digestive Health Care Center, Basel, Switzerland; Department of General, Visceral and Thoracic Surgery, University Medical Center Hamburg-Eppendorf, Hamburg, Germany; Department of General, Visceral and Thoracic Surgery, University Medical Center Hamburg-Eppendorf, Hamburg, Germany; Department of General, Visceral and Thoracic Surgery, University Medical Center Hamburg-Eppendorf, Hamburg, Germany; Department of General, Visceral and Thoracic Surgery, University Medical Center Hamburg-Eppendorf, Hamburg, Germany; Department of General, Visceral and Thoracic Surgery, University Medical Center Hamburg-Eppendorf, Hamburg, Germany; Department of General, Visceral and Thoracic Surgery, University Medical Center Hamburg-Eppendorf, Hamburg, Germany

The Clavien–Dindo classification (CDC) is the most widely used tool for reporting postoperative complications in randomized clinical trials (RCTs) in pancreatic surgery^1^. Although the CDC provides a standardized framework, there is still noticeable variability in how major complications (MCs) are defined. Different thresholds (CDC ≥ II in one *versus* CDC ≥ IIIb in another study) raise concerns regarding the comparability of RCTs. Such inconsistency in the definition of MCs represents a critical scientific issue, because it undermines the quality of systematic reviews, meta-analyses, and clinical guidelines. This systematic review investigated differences in the definition of MCs in RCTs in pancreatic surgery when applying the CDC.

This systematic review was conducted according to the Cochrane Handbook for Systematic Reviews and Interventions and is reported following the PRISMA guidelines. A literature search was performed using the Evidence Map of Pancreatic Surgery platform (https://map.eviglance.com/maps/8?view=map) up to 27 October 2024. The inclusion criterion was the definition of MCs according to the CDC. The exclusion criteria were the absence of a definition of MCs in the methods, missing reporting of MCs in the results, articles not written in English, and studies published before the CDC was introduced (that is, before 2004). The following details were extracted: study authors, publication year, sample size, definition of MCs, primary endpoint, and the number of patients. Data were analysed using SPSS^®^ (version 29; IBM, Armonk, NY, USA).

Of 1066 studies on pancreatic surgery, 366 were identified as RCTs by the Evidence Map of Pancreatic Surgery platform. Sixty-six studies defined MCs according to the CDC and were included in the analysis (*Table S1*). Most studies (52, 78.8%) defined MCs as CDC ≥ III. Five studies (7.6%) defined MCs as only CDC III–IV. Four studies (6.1%) defined MCs as CDC ≥ IIIb, and one study (1.5%) defined MCs as CDC > IIIb. CDC ≥ II and CDC II–IV were both used twice (3.0%) to define MCs (*Table S1*).

There were 20 different ways of reporting postoperative complications according to the CDC. Most studies (24, 36.4%) reported only CDC ≥ III. Nine studies (13.6%) reported postoperative complications without distinguishing CDC IIIa from CDC IIIb or CDC IVa from CDC IVb. Only 12 (18.2%) of the 66 included studies reported all CDC grades. The remaining studies (23, 34.8%) were distributed across the 17 observed CDC variations for reporting postoperative complications (*Fig. 1*).

**Fig. 1:**
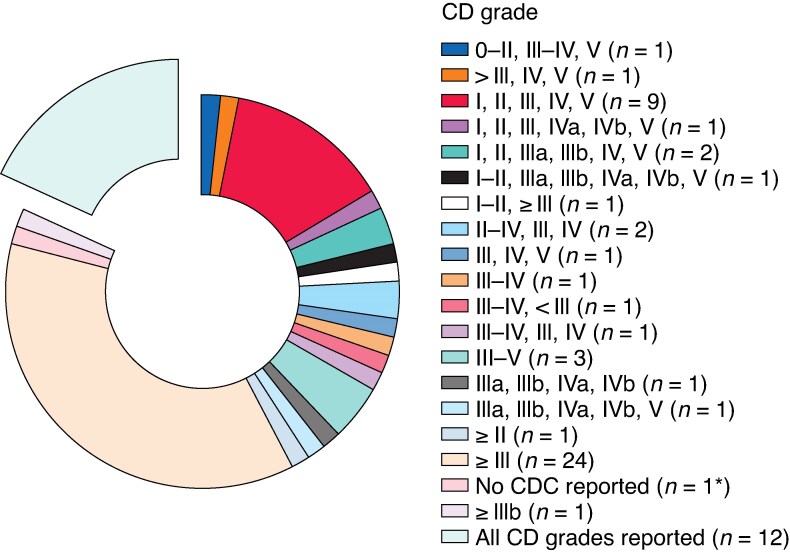
Observed variations in CDC reporting in the 66 studies included in the analysis *MCs were defined in the methods without reporting of MCs or any CD grades in the results. CDC, Clavien–Dindo classification; CD, Clavien–Dindo; MCs, major complications.

The MC variations showed inconsistency across the included RCTs. Despite the rigorous methodology described, most studies developed different definitions of MCs and unique approaches to report postoperative complications. This raised reasonable concern about how results of RCTs are being interpreted.

MCs are a valid clinical outcome because they are linked to severe complications that affect healthcare quality and cost^2,3^. However, inconsistencies in the definition of MCs undermine objective and reproducible data interpretation. The failure to objectively determine whether an intervention reduces the rate of postoperative complications hampers improvements in patient care and increases healthcare costs^4,5^. The next issue was the incomplete reporting of CDC grades. The tendency to report postoperative complications using different CDC modifications often made it difficult to distinguish between CDC IIIa and CDC IIIb, or CDC IVa and CDC IVb, because they were frequently reported together. The inability to differentiate between the CDC grades hinders the interpretation of outcomes and data synthesis.

Consistent reporting of postoperative complications in RCTs is crucial for providing evidence-based guidance. Adopting a universal definition of MCs can enhance objectivity.

## Supplementary Material

zraf103_Supplementary_Data

## Data Availability

All data used is provided in the manuscript or as *Supplementary material*.

## References

[1] Dindo D, Demartines N, Clavien PA. Classification of surgical complications: a new proposal with evaluation in a cohort of 6336 patients and results of a survey. Ann Surg 2004;240:205–213 15273542 10.1097/01.sla.0000133083.54934.ae PMC1360123

[2] Yuan P, Wu Z, Li Z, Bu Z, Wu A, Wu X et al Impact of postoperative major complications on long-term survival after radical resection of gastric cancer. BMC Cancer 2019;19:833 31443699 10.1186/s12885-019-6024-3 PMC6708212

[3] Cienfuegos JA, Baixauli J, Beorlegui C, Ortega PM, Granero L, Zozaya G et al The impact of major postoperative complications on long-term outcomes following curative resection of colon cancer. Int J Surg 2018;52:303–308 29530829 10.1016/j.ijsu.2018.03.001

[4] Vonlanthen R, Slankamenac K, Breitenstein S, Puhan MA, Muller MK, Hahnloser D et al The impact of complications on costs of major surgical procedures: a cost analysis of 1200 patients. Ann Surg 2011;254:907–913 21562405 10.1097/SLA.0b013e31821d4a43

[5] Téoule P, Bartel F, Birgin E, Rückert F, Wilhelm TJ. The Clavien–Dindo classification in pancreatic surgery: a clinical and economic validation. J Invest Surg 2019;32:314–320 29336625 10.1080/08941939.2017.1420837

