# Supplementary material for: Reporting of major complications in randomized clinical trials in pancreatic surgery according to Clavien–Dindo classification
Source: BJS Open. 2025 Sep 9;9(5):zraf103. doi: 10.1093/bjsopen/zraf103 (PMC12419532; doi:10.1093/bjsopen/zraf103)
Supplement: zraf103_Supplementary_Data [file zraf103_supplementary_data.docx]

**Reporting of Major Complications in Randomized Controlled Trials in Pancreatic Surgery According to Clavien-Dindo Classification**

Amila Cizmic, MD^1*^, Laetitia Hampe, MD^1*^, Philipp A. Wise, MD^2^, Pascal Probst, MD^3^, Markus K. Muller, MD^3^, Christoph Kuemmerli, MD^4^, Philip C. Müller, MD^4^, Jan Bardenhagen, MD^1^, Anna Nießen, MD^1^, Faik G. Uzunoglu, MD^1^, Jakob Izbicki, MD^1^, Thilo Hackert, MD^1^, Felix Nickel, MD, MME^1^

^1^ Department of General, Visceral and Thoracic Surgery, University Medical Center Hamburg-Eppendorf, Martinistraße 52, 20251 Hamburg, Germany

^2^ Department of Neuroradiology at the Neurology Center, Heidelberg University Hospital, Im Neuenheimer Feld 420, 69120 Heidelberg, Germany

^3^ Department of Surgery, Cantonal Hospital Thurgau, Frauenfeld, Switzerland

^4^ Clarunis University Digestive Health Care Center, Basel, Switzerland

* These authors contributed equally to the manuscript

**Corresponding Author:**

Felix Nickel, MME

Department of General, Visceral and Thoracic Surgery

University Medical Center Hamburg-Eppendorf

Martinistraße 52

20246 Hamburg, Germany

E-mail address: f.nickel@uke.de

**Supplementary Materials - Index**

| **Supplementary Results** |  | |
| --- | --- | --- |
| **Table 1** Basic characteristics of the RCTs included in the study (n = 66) | | *page 2* |

**Supplementary Results**

**Supplementary Material 1**

**Table 1** Basic characteristics of the RCTs included in the study (n = 66)

| First author | Year | Total n of patients | All CDC reported | MC definition* | Comments** on the CDC reporting |
| --- | --- | --- | --- | --- | --- |
| Klotz (1) | 2024 | 81 | no | ≥ III*** | only MC reported**** |
| Yoon (2) | 2024 | 235 | no | ≥ III | only MC reported |
| Kant (3) | 2024 | 70 | no | ≥ III | only MC reported |
| Chen (4) | 2024 | 265 | no | ≥ III | IIIa/b, IVa/b reported together |
| Gaujoux (5) | 2024 | 651 | no | ≥ III | only MC reported |
| Liu (6) | 2024 | 164 | no | ≥ III | only MC reported |
| van Bodegraven (7) | 2024 | 275 | no | ≥ III | only MC reported |
| Korrel (8) | 2023 | 258 | no | ≥ III | only MC reported |
| Serradilla-Martín (9) | 2023 | 64 | no | ≥ III | only MC reported |
| Smits (10) | 2022 | 1748 | no | ≥ III | only MC reported |
| Balzano (11) | 2022 | 61 | no | ≥ III | IIIa and IIIb reported together |
| Weyhe (12) | 2022 | 56 | no | III-IV | III-IV and < III |
| Dai (13) | 2022 | 312 | yes | II-IV | - |
| Korrel (14) | 2021 | 104 | no | ≥ III | only MC reported |
| Landoni (15) | 2021 | 145 | no | ≥ III | only MC reported |
| Mungroop (16) | 2021 | 247 | no | ≥ III | only MC reported |
| Uranues (17) | 2021 | 315 | no | ≥ III | only MC reported |
| Wang (18) | 2021 | 594 | no | ≥ III | only MC reported |
| Wennerblom (19) | 2021 | 106 | yes | ≥ IIIb | - |
| Andrianello (20) | 2020 | 72 | yes | ≥ III | - |
| Bergeat (21) | 2020 | 111 | yes | ≥ II | - |
| Björnsson (22) | 2020 | 58 | no | ≥ III | IIIa/b, IVa/b, and V reported |
| Dai (23) | 2020 | 144 | no | II-IV | II-IV, IIIa/b and IVa/b reported together |
| De Pastena (24) | 2020 | 190 | no | ≥ III | only MC reported |
| Liu (25) | 2020 | 231 | no | ≥ III | I-II and ≥ III reported |
| Park (26) | 2020 | 54 | no | ≥ III | only MC reported |
| Singh (27) | 2020 | 40 | no | III-IV | II/IV, IIIa/b, and IVa/b reported |
| Tarvainen (28) | 2020 | 126 | no | ≥ IIIb | only MC reported |
| Tumas (29) | 2020 | 70 | yes | ≥ III | - |
| Yamaguchi (30) | 2020 | 52 | no | ≥ III | IIIa, IIIb, IVa, IVb, and V reported |
| De Rooij (31) | 2019 | 108 | no | ≥ III | only MC reported |
| Antila (32) | 2019 | 47 | no | ≥ III | IIIa/b, IVa/b reported together |
| Ausania (33) | 2019 | 40 | no | III-IV | only MC reported |
| Busquets (34) | 2019 | 80 | no | ≥ III | only MC reported |
| Dembinski (35) | 2019 | 141 | no | ≥ III | 0-II, III-IV, and V reported |
| Hirono (36) | 2019 | 224 | yes | ≥ III | - |
| Kondo (37) | 2019 | 120 | no | ≥ III | IIIa/b, IVa/b reported together |
| Kwon (38) | 2019 | 124 | no | ≥ III | IIIa/b, IVa/b reported together |
| Sabater (39) | 2019 | 153 | no | ≥ III | only MC reported |
| van Hilst (40) | 2019 | 99 | no | ≥ III | IIIa, IIIb, IVa, and IVb reported |
| van Hilst (41) | 2019 | 38 | no | ≥ III | ≥ III, IV, and V reported |
| Cecka (42) | 2018 | 222 | no | ≥ III | only MC reported |
| El Nakeeb (43) | 2018 | 104 | no | ≥ III | IIIa/b, IVa/b reported together |
| Poves (44) | 2018 | 66 | no | ≥ III | I and II reported together |
| Senda (45) | 2018 | 120 | no | III-IV | IIIa/b and IVa/b reported together |
| Takeda (46) | 2018 | 50 | no | ≥ II | only MC reported |
| Yamamoto (47) | 2018 | 82 | no | ≥ III | only MC reported |
| Chen (48) | 2017 | 100 | no | ≥ III | only MC reported |
| Fujieda (49) | 2017 | 68 | no | ≥ III | only MC reported |
| Bai (50) | 2016 | 132 | no | ≥ III | IVa and IVb reported together |
| Grant (51) | 2016 | 330 | no | ≥ III | IIIa/b, IVa/b reported together |
| Kawai (52) | 2016 | 123 | yes | ≥ III | - |
| Laaninen (53) | 2016 | 62 | no | III-IV | III-IV, IIIa/b and IVa/b reported together |
| El Nakeeb (54) | 2015 | 107 | no | ≥ IIIb | IIIa/b, IVa/b reported together |
| El Nakeeb (55) | 2014 | 90 | no | > IIIb° | IIIa/b, IVa/b reported together |
| Zhu (56) | 2014 | 68 | yes | ≥ III | - |
| Figueras (57) | 2013 | 123 | no | ≥ III | only MC reported |
| Martin (58) | 2013 | 57 | yes | ≥ III | - |
| Topal (59) | 2013 | 329 | no | ≥ III | only MC reported |
| Uzunoglu (60) | 2013 | 89 | yes | ≥ IIIb | - |
| Zhu (61) | 2013 | 76 | yes | ≥ III | - |
| Frozanpor (62) | 2012 | 58 | no | ≥ III | IVa and IVb reported together |
| Motoi (63) | 2012 | 93 | no | ≥ III | only MC reported |
| Berger (64) | 2009 | 197 | no | ≥ III | only MC reported |
| Tien (65) | 2009 | 247 | yes | ≥ III | - |
| Winter (66) | 2006 | 234 | no | ≥ III | only MC reported |

RCT, randomized controlled trial; CDC, Clavien-Dindo Classification; n, number; MC major complications; POPF, postoperative pancreatic fistula; *, according to CDC; **, no comments for RCTs that reported all CDC (marked with "-"); ***, MC defined in methods but not reported in results; ****, only MC reported without reporting individual CDC postoperative complications; °, MC definition inconsistent (MC definition in Methods CDC > III and Results MC presented as > IIIb).

**References of the includes studies**

1. Klotz R, Mihaljevic AL, Kulu Y, Sander A, Klose C, Behnisch R, et al. Robotic versus open partial pancreatoduodenectomy (EUROPA): a randomised controlled stage 2b trial. Lancet Reg Health Eur. 2024;39:100864.

2. Yoon Y-S, Lee W, Kang CM, Hong T, Shin SH, Lee JW, et al. Laparoscopic versus open pancreatoduodenectomy for periampullary tumors: A randomized clinical trial. International Journal of Surgery. 9900:10.1097/JS9.0000000000002035.

3. Kant K, Ahmed Z, Dama R, Karunakaran M, Arora P, Rebala P, et al. Does perioperative hydrocortisone or indomethacin improve pancreatoduodenectomy outcomes? A triple arm, randomized placebo-controlled trial. Ann Hepatobiliary Pancreat Surg. 2024;28(3):350-7.

4. Chen H, Wang Y, Jiang K, Xu Z, Jiang Y, Wu Z, et al. The Effect of Perioperative Dexamethasone on Postoperative Complications After Pancreaticoduodenectomy: A Multicenter Randomized Controlled Trial. Ann Surg. 2024;280(2):222-8.

5. Gaujoux S, Regimbeau J-M, Piessen G, Truant S, Foissac F, Barbier L, et al. Somatostatin Versus Octreotide for Prevention of Postoperative Pancreatic Fistula: The PREFIPS Randomized Clinical Trial: A FRENCH 007—ACHBT Study. Annals of Surgery. 2024;280(2):179-87.

6. Liu Q, Li M, Gao Y, Jiang T, Han B, Zhao G, et al. Effect of robotic versus open pancreaticoduodenectomy on postoperative length of hospital stay and complications for pancreatic head or periampullary tumours: a multicentre, open-label randomised controlled trial. Lancet Gastroenterol Hepatol. 2024;9(5):428-37.

7. van Bodegraven EA, Balduzzi A, van Ramshorst TME, Malleo G, Vissers FL, van Hilst J, et al. Prophylactic abdominal drainage after distal pancreatectomy (PANDORINA): an international, multicentre, open-label, randomised controlled, non-inferiority trial. Lancet Gastroenterol Hepatol. 2024;9(5):438-47.

8. Korrel M, Jones LR, van Hilst J, Balzano G, Björnsson B, Boggi U, et al. Minimally invasive versus open distal pancreatectomy for resectable pancreatic cancer (DIPLOMA): an international randomised non-inferiority trial. Lancet Reg Health Eur. 2023;31:100673.

9. Serradilla-Martín M, Paterna-López S, Palomares-Cano A, Cantalejo-Díaz M, Abadía-Forcén T, Gutiérrez-Díez ML, et al. Polyethylene glycol-coated haemostatic patch for prevention of clinically relevant postoperative pancreatic fistula after pancreatoduodenectomy: randomized clinical trial. BJS Open. 2023;7(2).

10. Smits FJ, Henry AC, Besselink MG, Busch OR, van Eijck CH, Arntz M, et al. Algorithm-based care versus usual care for the early recognition and management of complications after pancreatic resection in the Netherlands: an open-label, nationwide, stepped-wedge cluster-randomised trial. Lancet. 2022;399(10338):1867-75.

11. Balzano G, Zerbi A, Aleotti F, Capretti G, Melzi R, Pecorelli N, et al. Total Pancreatectomy With Islet Autotransplantation as an Alternative to High-risk Pancreatojejunostomy After Pancreaticoduodenectomy: A Prospective Randomized Trial. Ann Surg. 2023;277(6):894-903.

12. Weyhe D, Obonyo D, Uslar V, Tabriz N. Effects of intensive physiotherapy on Quality of Life (QoL) after pancreatic cancer resection: a randomized controlled trial. BMC Cancer. 2022;22(1):520.

13. Dai M, Liu Q, Xing C, Tian X, Cao F, Tang W, et al. Early Drain Removal is Safe in Patients With Low or Intermediate Risk of Pancreatic Fistula After Pancreaticoduodenectomy: A Multicenter, Randomized Controlled Trial. Ann Surg. 2022;275(2):e307-e14.

14. Korrel M, Roelofs A, van Hilst J, Busch OR, Daams F, Festen S, et al. Long-Term Quality of Life after Minimally Invasive vs Open Distal Pancreatectomy in the LEOPARD Randomized Trial. J Am Coll Surg. 2021;233(6):730-9.e9.

15. Landoni L, De Pastena M, Fontana M, Malleo G, Esposito A, Casetti L, et al. A randomized controlled trial of stapled versus ultrasonic transection in distal pancreatectomy. Surg Endosc. 2022;36(6):4033-41.

16. Mungroop TH, van der Heijde N, Busch OR, de Hingh IH, Scheepers JJ, Dijkgraaf MG, et al. Randomized clinical trial and meta-analysis of the impact of a fibrin sealant patch on pancreatic fistula after distal pancreatectomy: CPR trial. BJS Open. 2021;5(3).

17. Uranues S, Fingerhut A, Belyaev O, Zerbi A, Boggi U, Hoffmann MW, et al. Clinical Impact of Stump Closure Reinforced With Hemopatch on the Prevention of Clinically Relevant Pancreatic Fistula After Distal Pancreatectomy: A Multicenter Randomized Trial. Ann Surg Open. 2021;2(1):e033.

18. Wang M, Li D, Chen R, Huang X, Li J, Liu Y, et al. Laparoscopic versus open pancreatoduodenectomy for pancreatic or periampullary tumours: a multicentre, open-label, randomised controlled trial. Lancet Gastroenterol Hepatol. 2021;6(6):438-47.

19. Wennerblom J, Ateeb Z, Jönsson C, Björnsson B, Tingstedt B, Williamsson C, et al. Reinforced versus standard stapler transection on postoperative pancreatic fistula in distal pancreatectomy: multicentre randomized clinical trial. Br J Surg. 2021;108(3):265-70.

20. Andrianello S, Marchegiani G, Malleo G, Masini G, Balduzzi A, Paiella S, et al. Pancreaticojejunostomy With Externalized Stent vs Pancreaticogastrostomy With Externalized Stent for Patients With High-Risk Pancreatic Anastomosis: A Single-Center, Phase 3, Randomized Clinical Trial. JAMA Surg. 2020;155(4):313-21.

21. Bergeat D, Merdrignac A, Robin F, Gaignard E, Rayar M, Meunier B, et al. Nasogastric Decompression vs No Decompression After Pancreaticoduodenectomy: The Randomized Clinical IPOD Trial. JAMA Surg. 2020;155(9):e202291.

22. Björnsson B, Larsson AL, Hjalmarsson C, Gasslander T, Sandström P. Comparison of the duration of hospital stay after laparoscopic or open distal pancreatectomy: randomized controlled trial. Br J Surg. 2020;107(10):1281-8.

23. Dai M, Liu Q, Xing C, Kleeff J, Liao Q, Guo J, et al. Early drain removal after major pancreatectomy reduces postoperative complications: a single-center, randomized, controlled trial. Journal of Pancreatology. 2020;3(2):93-100.

24. De Pastena M, Marchegiani G, Paiella S, Fontana M, Esposito A, Casetti L, et al. Use of an intraoperative wound protector to prevent surgical-site infection after pancreatoduodenectomy: randomized clinical trial. Br J Surg. 2020;107(9):1107-13.

25. Liu Q, Zhao Z, Gao Y, Zhao G, Jiang N, Lau WY, et al. Novel Technique for Single-Layer Pancreatojejunostomy is Not Inferior to Modified Blumgart Anastomosis in Robotic Pancreatoduodenectomy: Results of a Randomized Controlled Trial. Ann Surg Oncol. 2021;28(4):2346-55.

26. Park Y, Ko JH, Kang DR, Lee JH, Hwang DW, Lee JH, et al. Effect of Flowable Thrombin-Containing Collagen-Based Hemostatic Matrix for Preventing Pancreatic Fistula after Pancreatectomy: A Randomized Clinical Trial. J Clin Med. 2020;9(10).

27. Singh H, Krishnamurthy G, Kumar H, Gorsi U, Kumar MP, Mandavdhare H, et al. Effect of bile duct clamping versus no clamping on surgical site infections in patients undergoing pancreaticoduodenectomy: a randomized controlled study. ANZ J Surg. 2020;90(7-8):1434-40.

28. Tarvainen T, Sirén J, Kokkola A, Sallinen V. Effect of Hydrocortisone vs Pasireotide on Pancreatic Surgery Complications in Patients With High Risk of Pancreatic Fistula: A Randomized Clinical Trial. JAMA Surg. 2020;155(4):291-8.

29. Tumas J, Jasiūnas E, Strupas K, Šileikis A. Effects of Immunonutrition on Comprehensive Complication Index in Patients Undergoing Pancreatoduodenectomy. Medicina (Kaunas). 2020;56(2).

30. Yamaguchi H, Kimura Y, Imamura M, Nagayama M, Ito T, Kyuno D, et al. Effect of Rikkunshito, a Traditional Japanese Herbal Medicine, on Delayed Gastric Emptying and Oral Dietary Intake After Pancreaticoduodenectomy: A Prospective, Randomized, Single-Center, Open-Labeled Study. Clin Exp Gastroenterol. 2020;13:577-87.

31. de Rooij T, van Hilst J, van Santvoort H, Boerma D, van den Boezem P, Daams F, et al. Minimally Invasive Versus Open Distal Pancreatectomy (LEOPARD): A Multicenter Patient-blinded Randomized Controlled Trial. Ann Surg. 2019;269(1):2-9.

32. Antila A, Siiki A, Sand J, Laukkarinen J. Perioperative hydrocortisone treatment reduces postoperative pancreatic fistula rate after open distal pancreatectomy. A randomized placebo-controlled trial. Pancreatology. 2019;19(5):786-92.

33. Ausania F, Senra P, Meléndez R, Caballeiro R, Ouviña R, Casal-Núñez E. Prehabilitation in patients undergoing pancreaticoduodenectomy: a randomized controlled trial. Rev Esp Enferm Dig. 2019;111(8):603-8.

34. Busquets J, Martín S, Fabregat J, Secanella L, Pelaez N, Ramos E. Randomized trial of two types of gastrojejunostomy after pancreatoduodenectomy and risk of delayed gastric emptying (PAUDA trial). Br J Surg. 2019;106(1):46-54.

35. Dembinski J, Mariette C, Tuech JJ, Mauvais F, Piessen G, Fuks D, et al. Early removal of intraperitoneal drainage after pancreatoduodenectomy in patients without postoperative fistula at POD3: Results of a randomized clinical trial. J Visc Surg. 2019;156(2):103-12.

36. Hirono S, Kawai M, Okada KI, Miyazawa M, Kitahata Y, Hayami S, et al. Modified Blumgart Mattress Suture Versus Conventional Interrupted Suture in Pancreaticojejunostomy During Pancreaticoduodenectomy: Randomized Controlled Trial. Ann Surg. 2019;269(2):243-51.

37. Kondo N, Uemura K, Nakagawa N, Okada K, Kuroda S, Sudo T, et al. A Multicenter, Randomized, Controlled Trial Comparing Reinforced Staplers with Bare Staplers During Distal Pancreatectomy (HiSCO-07 Trial). Ann Surg Oncol. 2019;26(5):1519-27.

38. Kwon J, Shin SH, Lee S, Park G, Park Y, Lee SJ, et al. The Effect of Fibrinogen/Thrombin-Coated Collagen Patch (TachoSil(®)) Application in Pancreaticojejunostomy for Prevention of Pancreatic Fistula After Pancreaticoduodenectomy: A Randomized Clinical Trial. World J Surg. 2019;43(12):3128-37.

39. Sabater L, Cugat E, Serrablo A, Suarez-Artacho G, Diez-Valladares L, Santoyo-Santoyo J, et al. Does the Artery-first Approach Improve the Rate of R0 Resection in Pancreatoduodenectomy?: A Multicenter, Randomized, Controlled Trial. Ann Surg. 2019;270(5):738-46.

40. van Hilst J, de Rooij T, Bosscha K, Brinkman DJ, van Dieren S, Dijkgraaf MG, et al. Laparoscopic versus open pancreatoduodenectomy for pancreatic or periampullary tumours (LEOPARD-2): a multicentre, patient-blinded, randomised controlled phase 2/3 trial. Lancet Gastroenterol Hepatol. 2019;4(3):199-207.

41. van Hilst J, Brinkman DJ, de Rooij T, van Dieren S, Gerhards MF, de Hingh IH, et al. The inflammatory response after laparoscopic and open pancreatoduodenectomy and the association with complications in a multicenter randomized controlled trial. HPB (Oxford). 2019;21(11):1453-61.

42. Čečka F, Jon B, Skalický P, Čermáková E, Neoral Č, Loveček M. Results of a randomized controlled trial comparing closed-suction drains versus passive gravity drains after pancreatic resection. Surgery. 2018;164(5):1057-63.

43. El Nakeeb A, ElGawalby A, M AA, Shehta A, Hamed H, El Refea M, et al. Efficacy of octreotide in the prevention of complications after pancreaticoduodenectomy in patients with soft pancreas and non-dilated pancreatic duct: A prospective randomized trial. Hepatobiliary Pancreat Dis Int. 2018;17(1):59-63.

44. Poves I, Burdío F, Morató O, Iglesias M, Radosevic A, Ilzarbe L, et al. Comparison of Perioperative Outcomes Between Laparoscopic and Open Approach for Pancreatoduodenectomy: The PADULAP Randomized Controlled Trial. Ann Surg. 2018;268(5):731-9.

45. Senda Y, Shimizu Y, Natsume S, Ito S, Komori K, Abe T, et al. Randomized clinical trial of duct-to-mucosa versus invagination pancreaticojejunostomy after pancreatoduodenectomy. Br J Surg. 2018;105(1):48-57.

46. Takeda Y, Mise Y, Ishizuka N, Harada S, Hayama B, Inoue Y, et al. Effect of early administration of coagulation factor XIII on fistula after pancreatic surgery: the FIPS randomized controlled trial. Langenbecks Arch Surg. 2018;403(8):933-40.

47. Yamamoto T, Satoi S, Fujii T, Yamada S, Yanagimoto H, Yamaki S, et al. Dual-center randomized clinical trial exploring the optimal duration of antimicrobial prophylaxis in patients undergoing pancreaticoduodenectomy following biliary drainage. Ann Gastroenterol Surg. 2018;2(6):442-50.

48. Chen S, Zhan Q, Jin JB, Wu ZC, Shi Y, Cheng DF, et al. Robot-assisted laparoscopic versus open middle pancreatectomy: short-term results of a randomized controlled trial. Surg Endosc. 2017;31(2):962-71.

49. Fujieda H, Yokoyama Y, Hirata A, Usui H, Sakatoku Y, Fukaya M, et al. Does Braun Anastomosis Have an Impact on the Incidence of Delayed Gastric Emptying and the Extent of Intragastric Bile Reflux Following Pancreatoduodenectomy? - A Randomized Controlled Study. Dig Surg. 2017;34(6):462-8.

50. Bai X, Zhang Q, Gao S, Lou J, Li G, Zhang Y, et al. Duct-to-Mucosa vs Invagination for Pancreaticojejunostomy after Pancreaticoduodenectomy: A Prospective, Randomized Controlled Trial from a Single Surgeon. J Am Coll Surg. 2016;222(1):10-8.

51. Grant F, Brennan MF, Allen PJ, DeMatteo RP, Kingham TP, D'Angelica M, et al. Prospective Randomized Controlled Trial of Liberal Vs Restricted Perioperative Fluid Management in Patients Undergoing Pancreatectomy. Ann Surg. 2016;264(4):591-8.

52. Kawai M, Hirono S, Okada K, Sho M, Nakajima Y, Eguchi H, et al. Randomized Controlled Trial of Pancreaticojejunostomy versus Stapler Closure of the Pancreatic Stump During Distal Pancreatectomy to Reduce Pancreatic Fistula. Ann Surg. 2016;264(1):180-7.

53. Laaninen M, Sand J, Nordback I, Vasama K, Laukkarinen J. Perioperative Hydrocortisone Reduces Major Complications After Pancreaticoduodenectomy: A Randomized Controlled Trial. Ann Surg. 2016;264(5):696-702.

54. El Nakeeb A, El Hemaly M, Askr W, Abd Ellatif M, Hamed H, Elghawalby A, et al. Comparative study between duct to mucosa and invagination pancreaticojejunostomy after pancreaticoduodenectomy: a prospective randomized study. Int J Surg. 2015;16(Pt A):1-6.

55. El Nakeeb A, Hamdy E, Sultan AM, Salah T, Askr W, Ezzat H, et al. Isolated Roux loop pancreaticojejunostomy versus pancreaticogastrostomy after pancreaticoduodenectomy: a prospective randomized study. HPB (Oxford). 2014;16(8):713-22.

56. Zhu X, Wu Y, Qiu Y, Jiang C, Ding Y. Comparative analysis of the efficacy and complications of nasojejunal and jejunostomy on patients undergoing pancreaticoduodenectomy. JPEN J Parenter Enteral Nutr. 2014;38(8):996-1002.

57. Figueras J, Sabater L, Planellas P, Muñoz-Forner E, Lopez-Ben S, Falgueras L, et al. Randomized clinical trial of pancreaticogastrostomy versus pancreaticojejunostomy on the rate and severity of pancreatic fistula after pancreaticoduodenectomy. Br J Surg. 2013;100(12):1597-605.

58. Martin I, Au K. Does fibrin glue sealant decrease the rate of anastomotic leak after a pancreaticoduodenectomy? Results of a prospective randomized trial. HPB (Oxford). 2013;15(8):561-6.

59. Topal B, Fieuws S, Aerts R, Weerts J, Feryn T, Roeyen G, et al. Pancreaticojejunostomy versus pancreaticogastrostomy reconstruction after pancreaticoduodenectomy for pancreatic or periampullary tumours: a multicentre randomised trial. Lancet Oncol. 2013;14(7):655-62.

60. Uzunoglu FG, Bockhorn M, Fink JA, Reeh M, Vettorazzi E, Gawad KA, et al. LigaSure™ vs. conventional dissection techniques in pancreatic surgery--a prospective randomised single-centre trial. J Gastrointest Surg. 2013;17(3):494-500.

61. Zhu X, Wu Y, Qiu Y, Jiang C, Ding Y. Effect of parenteral fish oil lipid emulsion in parenteral nutrition supplementation combined with enteral nutrition support in patients undergoing pancreaticoduodenectomy. JPEN J Parenter Enteral Nutr. 2013;37(2):236-42.

62. Frozanpor F, Lundell L, Segersvärd R, Arnelo U. The effect of prophylactic transpapillary pancreatic stent insertion on clinically significant leak rate following distal pancreatectomy: results of a prospective controlled clinical trial. Ann Surg. 2012;255(6):1032-6.

63. Motoi F, Egawa S, Rikiyama T, Katayose Y, Unno M. Randomized clinical trial of external stent drainage of the pancreatic duct to reduce postoperative pancreatic fistula after pancreaticojejunostomy. Br J Surg. 2012;99(4):524-31.

64. Berger AC, Howard TJ, Kennedy EP, Sauter PK, Bower-Cherry M, Dutkevitch S, et al. Does type of pancreaticojejunostomy after pancreaticoduodenectomy decrease rate of pancreatic fistula? A randomized, prospective, dual-institution trial. J Am Coll Surg. 2009;208(5):738-47; discussion 47-9.

65. Tien YW, Yang CY, Wu YM, Hu RH, Lee PH. Enteral nutrition and biliopancreatic diversion effectively minimize impacts of gastroparesis after pancreaticoduodenectomy. J Gastrointest Surg. 2009;13(5):929-37.

66. Winter JM, Cameron JL, Campbell KA, Chang DC, Riall TS, Schulick RD, et al. Does pancreatic duct stenting decrease the rate of pancreatic fistula following pancreaticoduodenectomy? Results of a prospective randomized trial. J Gastrointest Surg. 2006;10(9):1280-90; discussion 90.
